# Vaccination in children with congenital heart disease: an observational study in a Beijing hospital

**DOI:** 10.1038/s41390-022-02344-w

**Published:** 2022-10-28

**Authors:** Xue-ying Zhou, Mi Yao, Jian-guang Qi, Zhen-nan Qi, Wei-lan Liang

**Affiliations:** 1 Department of General Practice, Peking University First Hospital, Beijing, China; 2 Department of General Practice, Peking University Health Science Center, Beijing, China; 3 Department of Pediatrics, Peking University First Hospital, No. 1, Xianmen Street, Xicheng District, 100034 Beijing, China

## Abstract

**Introduction:**

Underimmunization of CHD children is a public health concern in China. This study aimed to analyze the vaccination status of CHD children to provide additional evidence on optimal vaccination strategies and to make suggestions to promote appropriate vaccination services for these children.

**Methods:**

This cross-sectional study evaluated 155 CHD children who received at least one vaccine at Peking University First Hospital. Vaccine-specific immunization rates were calculated. A telephone questionnaire survey was conducted that covered the following: the prognosis, reasons for delayed vaccinations and getting vaccination in the hospital. All statistical analyses were performed using the SPSS version 22 software.

**Results:**

The left-to-right shunt group involved 138 children, while the other type CHD group involved 17. The vaccination rate was the highest for MPSV-AC (87.1%) and the lowest for DTaP (40.1%). The most frequent reason for vaccination in the hospital was refusal from community health centers (61.5%). No participant reported vaccine-related adverse effects.

**Conclusions:**

The age-appropriate vaccine-specific immunization rates in CHD children are low, with the lowest for DTaP. Refusal of community health centers was the primary reason. Our findings support that clinically stable CHD children may be safely vaccinated on a schedule similar to that of ordinary children in China.

**Impact:**

- From our investigation, we found that the age-appropriate vaccine-specific immunization rates in children with CHD in China are low, with the lowest for diphtheria and tetanus toxoid and acellular pertussis.
- Refusal of community health centers to vaccinate was the primary reason for the low rates.
- We believe our study provides additional evidence on optimal vaccination strategies for children with CHD and it can be used to develop strategies to promote appropriate vaccination services for these children.

## Introduction

Congenital heart disease (CHD) is the most common cause of major congenital anomalies. CHD is prevalent in approximately 8 per 1000 live births worldwide.^1^ In China, a recent study in Beijing showed that CHD is prevalent in 8.2/1000 births, and approximately 150,000 children are born with CHD annually.^2^ Medical advances have improved the life expectancy of children with CHD, and it is now similar to that of the general population. CHD mortality has markedly decreased, and deaths have decreased in both infants and adults.^3^

Scheduled immunization programs for children are a crucial preventative measure to decrease morbidity and mortality from infectious diseases. Children with CHD are susceptible to infectious diseases that progress to serious complications. Particularly, CHD is associated with more severe complications and higher mortality from infectious diseases.^4–6^ Therefore, the vaccination of children with CHD is equally important as or more important than that of the general population for preventing infectious diseases.

However, underimmunization of children with CHD is a public health concern in China. Some studies have found low age-specific vaccination rates in children with CHD, and vaccination of these children is often delayed or missed.^7–12^ There are no studies that have clearly investigated the reasons for the high underimmunization (not up-to-date) rates in children with CHD. In addition, some community health centers refuse vaccinating children with CHD due to safety concerns. These children then have to go to tertiary hospitals to receive their vaccines. Large-scale studies are needed to further demonstrate the safety of vaccination in these children and whether these children can receive vaccines in the community.

As such, the primary objective of this study was to examine the rates of scheduled vaccines in children with CHD and to assess the safety of these vaccinations, particularly when given in community health centers. The secondary objective was to explore the reasons for delayed immunization to facilitate the formation of more effective vaccination strategies for children with CHD in China. We hypothesized that the rate of up-to-date vaccinations is lower in children with CHD than that in the general population of children.

## Methods

### Study design and patients

This cross-sectional study evaluated children with CHD who had received at least one vaccine at Peking University First Hospital (PKUFH) between February 7, 2002 and September 11, 2017. Vaccination information was accessed from the Beijing Immunization Planning Information Management System. PKUFH is a teaching hospital of Peking University that caters to approximately 200,000 outpatients annually and is generally representative of comprehensive tertiary hospitals in Beijing. The exclusion criteria were the absence of verifiable vaccination and medical records. The patients were divided into two groups based on the CHD type. The left-to-right shunt CHD group involved patients with patent ductus arteriosus (PDA), atrial septal defect (ASD), patent foramen ovale (PFO), ventricular septal defect (VSD), and a combination of two or three defect types mentioned above. The other CHD type group involved patients with pulmonary stenosis, tetralogy of Fallot, and other types.

**Fig. 1:**
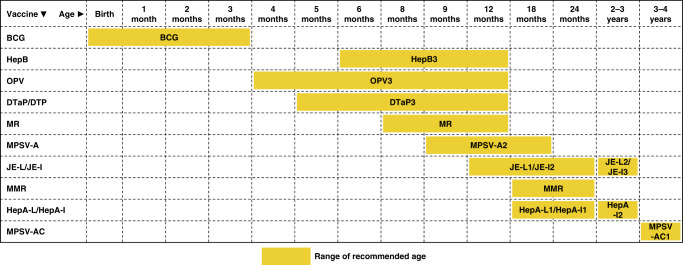
Age-specific vaccine recommendations according to the Scheduled Routine Vaccination Rates Monitoring Program in Beijing. Each bar refers to a timeline through which a certain vaccine is supposed age-appropriate according to the Scheduled Routine Vaccination Rates Monitoring Program in Beijing.

Baseline information of these children, including age, sex, and disease type, was obtained from the electronic medical charts. We then conducted a telephone questionnaire survey as follow up, from February to March 2018. The survey covered the following aspects: the prognosis of CHD, factors inducing delayed vaccinations, and reasons for getting vaccination in the hospital instead of community health service centers. The routine vaccines for children in China are BCG, hepatitis B (HepB), diphtheria and tetanus toxoid and acellular pertussis (DTaP), oral poliovirus (OPV), measles vaccine/measles and rubella vaccine (MV/MR), measles, mumps, and rubella (MMR), hepatitis A virus (HAV), meningococcal polysaccharide A vaccine (MPSV-A), Japanese encephalitis vaccine (JEV). The recommended age for vaccination according to the Scheduled Routine Vaccination Rates Monitoring Program of Beijing published in 2009 is shown in Fig. 1.

### Statistical analysis

The vaccine-specific immunization rates of children at the recommended age were calculated. Demographic characteristics and vaccination rates were summarized using descriptive statistics (median and interquartile range (IQR) for continuous variables; percentage (rate) for categorical variables) and compared between different CHD type groups. Differences between groups were tested for statistical significance using the Pearson *χ*^2^ or *Z*-tests as appropriate. All statistical analyses were performed using the SPSS version 22 software. An *α* value of 0.05 was considered statistically significant. The process of data calculation and analysis was done from March 2018 to June 2019.

## Results

### Population characteristics

In total, 155 children aged between 0.4 years and 15.6 years were included in the analysis. Of them, 99 were girls (63.9%) and 56 were boys (36.1%). The median (IQR) age was 5.1 years (3.2–8.5 years). Sixteen children were born premature or had low body weight, and four children had chromosomal diseases or other birth defects. The demographic characteristics of the children are presented in Table 1. Overall, 138 children were categorized to the left-to-right shunt defect group, including 21 children with PDA, 43 children with ASD, 9 children with PFO, 42 children with VSD, and 23 children with combined left to right shunt congenital heart defects (12 children with ASD combined VSD, 7 children with ASD combined PDA, 3 children with VSD combined PDA, 1 child with ASD combined VSD and PDA). 17 children were categorized to the other CHD type group, including VSD combined with coronary pulmonary artery fistula, pulmonary stenosis, tetralogy of Fallot, and aortic stenosis and the lesion complexity was more severe.

**Table 1:** Demographic characteristics of the participants (*n* = 155).

Demographics	PDA (*n* = 21)	ASD (*n* = 43)	PFO (*n* = 9)	VSD (*n* = 42)	Combined left-to-right shunt CHD defects (*n* = 23)	Other types (*n* = 17)	*P* value
Sex, No.							
Boy	8	14	5	14	5	10	0.173
Girl	13	29	4	28	18	7	
Age, years							
<1	0	4	2	1	3	1	
1–6	7	22	5	19	11	11	0.140
>6	14	17	2	22	9	5	

### Vaccination status

The vaccination rate at the recommended age varied among the vaccines. The rate was the highest for meningococcal polysaccharide A and C (MPSV-AC) at 87.1%, whereas it was the lowest for DTaP at only 40.1%. The rates of the first shot of JEV (JEV-1) and BCG vaccine were 85.9% and 85.7%, respectively. Except for BCG, JEV-1, and MPSV-AC, the vaccination rates at the recommended age were all below the standard recommendation in Beijing (Table 2).

**Table 2:** Age-recommended vaccination rates.

Vaccines	Vaccination rate in CHD children at recommended age (%, (*N*))	Guideline-recommended vaccination rate in Beijing (%)	*Z*	*P* value
BCG	85.7 (132)	≥90	−1.7993	>0.05
HepB	72.9 (105)	≥95	−11.9457	<0.01
OPV	78.2 (115)	≥98	−17.1711	<0.01
DTP/DTaP	40.1 (59)	≥95	−30.5046	<0.01
MV/MR	64.1 (93)	≥98	−29.4509	<0.01
JE-L1/JE-I2	85.9 (116)	≥90	−1.7903	>0.05
MPSV-A2	75.4 (104)	≥90	−6.0868	<0.01
MMR	67.7 (86)	≥90	−8.0879	<0.01
Hep-A1	73.2 (93)	≥90	−6.5789	<0.01
JE-L2/JE-I3	83.5 (101)	≥90	−2.5578	<0.05
MPSV-AC1	87.1 (88)	≥90	−1.0385	>0.05

*BCG* Bacillus Calmette-Guérin, *HepB* hepatitis B vaccine, *OPV* oral poliovirus vaccine, *DTP/DTaP* diphtheria and tetanus toxoids and pertussis vaccine/diphtheria and tetanus toxoids and acellular pertussis vaccine, *MV/MR* measles vaccine/measles and rubella vaccine, *JE-L/JE-I* Japanese encephalitis live virus vaccine/Japanese encephalitis inactivated virus vaccine, *JE-L1/JE-I2* the first shot of Japanese encephalitis live virus vaccine/the second shot of Japanese encephalitis inactivated virus vaccine, *MPSV-A2* the second shot of meningococcal polysaccharide A vaccine, *MMR* measles, mumps and rubella vaccine, *HepA-L/HepA-I* hepatitis A live virus vaccine/hepatitis A inactivated virus vaccine, *Hep-A1* the first shot of hepatitis A virus vaccine, *HepA-L1/HepA-I1* the first shot of hepatitis A live virus vaccine/inactivated virus vaccine, *JE-L2/JE-I3* the second shot of Japanese encephalitis live virus vaccine/the third shot of Japanese encephalitis inactivated virus vaccine, *HepA-I2* the second shot of hepatitis A inactivated virus vaccine, *MPSV-AC1* the first shot of meningococcal polysaccharide A and C vaccine.

All children were evaluated before each vaccination and had no manifestations of heart failure or developmental problems. There were no recorded serious side effects observed or reported by parents after vaccination.

The vaccination rate at the recommended age for all vaccines was higher in the left-to-right shunt CHD group compared with other type CHD group (Table 3).

**Table 3:** Vaccination rates of different CHD type groups.

Vaccines^a^	Vaccinated as recommended or not	Left-to-right shunt CHD						Children with other type CHD, No. (%)
		PDA, No.	ASD, No.	PFO^b^, No.	VSD, No.	Combination, No.	Total, No. (%)	
BCG	Yes	16	40	8	37	21	122 (89.1)	10 (58.8)
	No	5	3	1	4	2	15 (10.9)	7 (41.2)
HepB	Yes	15	28	7	28	20	98 (76.6)	7 (43.8)
	No	6	9	2	12	1	30 (23.4)	9 (56.3)
OPV	Yes	14	35	8	30	20	107 (82.3)	8 (47.1)
	No	7	5	1	10	0	23 (17.7)	9 (52.9)
DTP/DTaP	Yes	11	17	6	10	12	56 (43.1)	3 (17.6)
	No	10	23	3	30	8	74 (56.9)	14 (82.4)
MR/MV	Yes	11	28	7	26	15	87 (67.4)	6 (37.5)
	No	10	13	1	13	5	42 (32.6)	10 (62.5)
JE-L1/JE-I2	Yes	18	32	6	35	16	107 (89.9)	9 (56.3)
	No	2	5	0	2	3	12 (10.1)	7 (43.8)
MPSV-A2	Yes,	16	29	7	25	19	96 (78.7)	8 (50.0)
	No	5	8	0	12	1	26 (21.3)	8 (50.0)
MMR	Yes	14	27	3	24	13	81 (71.1)	5 (38.5)
	No	4	9	2	13	5	33 (28.9)	8 (61.5)
HepA-L1/HepA-I1	Yes	14	29	5	27	14	89 (77.4)	4 (33.3)
	No	5	7	0	10	4	26 (22.6)	8 (66.4)
JE-L2/JE-I3	Yes	17	29	4	32	13	95 (87.2)	6 (50.0)
	No	2	5	0	5	2	14 (12.8)	6 (50.0)
HepA-I2	Yes	15	27	5	33	14	94 (87.9)	7 (58.3)
	No	2	5	0	4	2	13 (12.1)	5 (41.7)
MPSV-AC1	Yes	13	26	2	30	12	83 (91.2)	5 (50.0)
	No	1	2	0	2	3	8 (8.8)	5 (50.0)

*BCG* Bacillus Calmette-Guérin, *HepB* hepatitis B vaccine, *OPV* oral poliovirus vaccine, *DTP/DTaP* diphtheria and tetanus toxoids and pertussis vaccine/diphtheria and tetanus toxoids and acellular pertussis vaccine, *MV/MR* measles vaccine/measles and rubella vaccine, *JE-L/JE-I* Japanese encephalitis live virus vaccine/Japanese encephalitis inactivated virus vaccine, *JE-L1/JE-I2* the first shot of Japanese encephalitis live virus vaccine/the second shot of Japanese encephalitis inactivated virus vaccine, *MPSV-A2* the second shot of meningococcal polysaccharide A vaccine, *MMR* measles, mumps and rubella vaccine, *HepA-L/HepA-I* hepatitis A live virus vaccine/hepatitis A inactivated virus vaccine, *Hep-A1* the first shot of hepatitis A virus vaccine, *HepA-L1/HepA-I1* the first shot of hepatitis A live virus vaccine/inactivated virus vaccine, *JE-L2/JE-I3* the second shot of Japanese encephalitis live virus vaccine/the third shot of Japanese encephalitis inactivated virus vaccine, *HepA-I2* the second shot of hepatitis A inactivated virus vaccine, *MPSV-AC1* the first shot of meningococcal polysaccharide A and C vaccine.

^a^The number of children vaccination records who cannot be traced in the system due to home addresses moving out of Beijing: BCG (1), HepB (5), OPV (1), DTP/DTaP (1), MR/MV (4), JE-L1/JE-I2 (9), MPSV-A2 (5), MMR (15), HepA-L1/HepA-I1 (12), JE-L2/JE-I3 (15), HepA-I2 (13), MPSV-AC1 (17). The number of children age who has not yet reached the time for some vaccination: HepB (6), OPV (7), DTP/DTaP (7), MR/MV (6), JE-L1/JE-I2 (11), MPSV-A2 (12), MMR (13), HepA-L1/HepA-I1 (16), JE-L2/JE-I3 (19), HepA-I2 (23), MPSV-AC1 (37). The last dose of vaccines in this table for multiple doses of vaccines was recorded.

^b^PFO classified as CHD is currently controversial. We observed that Chinese children diagnosed with PFO were delayed for vaccination, so we counted PFO by left-to-right shunt CHD in our study.

### Reasons for being vaccinated in the hospital

Of the 155 cases, 104 cases were successfully contacted by telephone, yielding a survey response rate of 67.1%. The most frequent reason for getting vaccination in the hospital was that community health service centers refused to do the vaccination for them (61.5%). Other reasons were “considering that vaccination in hospital was safer” (16.3%) and “living closer to the hospital (7.7%)” (Table 4).

**Table 4:** Reasons for vaccination in the hospital by CHD type.

Reasons	PDA, No.	ASD, No.	PFO, No.	VSD, No.	Combined left-to-right shunt CHD, No.	Other types, No.	Total, No.
Rejected by community health center because of CHD	6	20	4	17	6	11	64
Hospital can provide regular checkup for them	2	0	1	0	0	0	3
Belief that the hospital is more trustworthy	4	4	1	3	2	3	17
Live near hospital	0	1	1	2	4	0	8
Unsatisfied with the community health center vaccination service	1	1	0	1	0	0	3
Complicated with other serious disease and rejected by community health center	2	0	0	2	0	0	4
Others	2	3	0	1	3	0	9

### Factors associated with delayed vaccination

The factors associated with delayed vaccination ranking were as follows: (1) doctors delayed the vaccination for these children based on their own judgment; (2) some vaccinations were delayed because of cold, fever, and diarrhea at the time of vaccination; (3) the local community health centers refused vaccination, and the family was not informed of where to get the vaccines; (4) hospitalization; (5) surgery, after which the vaccination was often delayed to ensure adequate observation; (6) immunoglobulin therapy.

### Long-term prognosis of children with CHD

At the time of follow-up, all children were stable, and most of the CHDs were spontaneously resolved or repaired via interventional therapy or surgery. No vaccine-related long-term sequelae was observed.

## Discussion

Scheduled immunization for children is an important basic public health service in China and has made great progress since the National Immunization Program was started in 1978. The vaccination rates of all scheduled vaccines are maintained above 90% in the general population of children.^13^ However, few studies in China have examined the vaccination rates in children with CHD, who are at higher risk of infectious diseases and worse outcomes. A study of 30 children with CHD conducted at a community health center in Shenzhen found that except for JEV and BCG vaccines, the age-appropriate vaccination rates of all other scheduled vaccines were lower than that of the general children population. Although the target rate is over 90%, the age-appropriate vaccination rate of MPSV and DTaP were only 65.1% and 60.1%, respectively.^7^ Another similar study of 36 children with CHD in Beijing also showed lower rates of vaccination at the recommended age except for BCG. In this study, the age-appropriate vaccination rate of HepB was only 68.2%; DTaP, 56.8%; and MPSV, 52.3%.^8^ However, these previous studies were all conducted in community health centers, and the children may have less severe CHD. Further, the studies had a relatively small sample size, and thus lack representativeness.

In our study, we also found that the age-recommended vaccination rates in children with CHD in Beijing were lower than those required by the Scheduled Routine Vaccination Rates Monitoring Program. The rates were low for all vaccines and also differed by vaccine. The vaccination rate for BCG was relatively high, probably because it is administered immediately after birth before CHD is diagnosed or because doctors are more confident to manage possible emergency events in tertiary hospitals with advanced devices than in a community health center. The rates of JEV and MPSV-AC vaccines were also higher than those of other vaccines, probably because these two vaccines are administered after 1 year of age, when the defects are resolved spontaneously or by surgery. The lowest rate of vaccination was for DTaP/DTP at 40.1%, and some children even missed the vaccine. The DTaP/DTP vaccine is commonly reported to be associated with adverse events, and thus doctors are more cautious and tend to delay or even avoid it in children with CHD.

We investigated the reasons for the delayed vaccination in these children through a telephone survey and found that the primary reason was refusal of vaccination from community health centers. Thus, the children needed to be taken to the hospital for vaccination. However, some children resided far from the hospital and were unable to immediately visit the hospital, delaying the vaccination. This is an important factor causing the low up-to-date vaccination rate and may be one of the important reasons for the lower vaccination rates in our study than those in community-based studies.^7,8^

Another important factor was that doctors tended to delay vaccination for these children mainly due to safety concerns. However, all 155 children in our study were clinically stable and did not experience serious vaccine-related adverse reactions or sequelae. Particularly, no serious adverse reactions were recorded in all vaccinations, even in children who were vaccinated as scheduled. This supports the safety of vaccination in these children. These children are more carefully assessed before vaccination, and vaccination is more cautiously performed. It is usually postponed when children present even the mildest conditions such as a cold. This would possibly explain the fewer adverse reactions. Further, although age-recommended vaccination rates for children with CHD were lower compared with the Guideline-recommended rate (more than 90%), a large proportion of them received vaccination as scheduled. For example, the vaccination rates of HBV, OPV, JEV, MPSV, and HAV at the recommended age were all above 70%.

In China, the primary care system for children still needs to be improved. Many general practitioners lack training and clinical experience for managing pediatric patients. Concerns about an inability to manage vaccine-related adverse effects may be the fundamental factor for refusal for vaccination of children with CHD in community health centers. In our investigation, even children with PFO, which rarely causes symptoms and is currently not considered a CHD, are also rejected by community health centers and are referred to secondary hospitals to receive vaccinations.

However, prior to our study, there were no formal recommendations or adequate safety evidence for vaccination of children with CHD in China. In our previous study, we found that community doctors considered that routine vaccination can be safely provided in children with VSD with a defect diameter ≤5 mm after ruling out heart failure and other contraindications while children whose VSD diameter was >5 mm were referred to the hospital for specialist opinions on vaccination.^13,14^ In Western countries, children with CHD are recommended to follow the routine immunization schedule, except for those with serious congestive heart failure or immune deficiency diseases.^15,16^ In China, only one expert consensus on vaccination of children with CHD was published earlier this year stating that CHD children with normal heart function can be vaccinated as ordinary children.^17^ The effect of the consensus on immunization of children with CHD requires further observation.

Our results showed that the age-recommended vaccination rate in children with left-to-right shunt CHD was higher than that in those with other CHD types. This is possible because a large proportion of children included in other CHD types (i.e., complex right-to-left shunt CHD) undergo surgery, and vaccinations are delayed by the attending physician at an average of 6 months to ensure an adequate post-operative observation. However, a previous study showed that the immunization function returned to normal 1–3 months after extracorporeal circulation surgery, and it is the optimal time to restart vaccination.^18^ This suggests a highly delayed vaccination for these children, which can lead to a higher risk of infection.

To our best knowledge, our study is the first to investigate the vaccination rate in children with CHD in China for a long period. In addition to a relatively large sample size, the vaccination status was analyzed in more detail. However, this study also has some limitations. The study was conducted in a single hospital in Beijing and only included children with nearly normal heart function. The vaccination rate for children with CHD in other parts of China, in those with unstable heart function, and those who undergo heart surgery still needs to be clarified.

## Conclusion

The age-recommended vaccination rate of children with CHD is lower than the standard recommended rate. The vaccination rate was the highest for MPSV-AC and the lowest for DTaP. The primary reasons for the lower rates are refusal of community health centers to perform the vaccination and physician-directed deferment of the vaccination. However, no participant reported vaccine-related adverse effects, thus supporting that clinically stable children with CHD may be safely vaccinated on a schedule similar to that of ordinary children. Although formal recommendations for the vaccination of children with CHD are starting to be developed in China, improving the health care system at the primary level and developing an efficient reference system are also needed to improve the vaccination rates in children with CHD.

## Data Availability

Materials described in the manuscript, including all relevant raw data, will be freely available by contacting the corresponding author to any researcher wishing to use them.
